# Supplementary material for: Effects of distancing and pattern of breathing on the filtering capability of commercial and custom-made facial masks: An in-vitro study
Source: PLoS One. 2021 Apr 22;16(4):e0250432. doi: 10.1371/journal.pone.0250432 (PMC8062003; doi:10.1371/journal.pone.0250432)
Supplement: S1 Table — (DOCX) [file pone.0250432.s001.docx]

**S1 Table.** Experimental dataset of percentage inhaled fraction as a function of type of device, breathing pattern and distance.

| **Device** | **Pattern of breathing** | **Distance (cm)** | **Measure 1** | **Measure 2** | **Measure 3** |
| --- | --- | --- | --- | --- | --- |
| Distancing alone | Normal | 40 | 57,1 | 57,6 | 57,9 |
| Surgical mask | Normal | 40 | 30,8 | 30,2 | 31,1 |
| FFP1 | Normal | 40 | 21,9 | 22,3 | 21,8 |
| FFP2 | Normal | 40 | 1,0 | 1,0 | 1,0 |
| FFP3 | Normal | 40 | 1,0 | 1,0 | 1,0 |
| FFP2 + Surgical mask | Normal | 40 | 1,0 | 1,0 | 1,0 |
| Malpositioned FFP2 | Normal | 40 | 12,3 | 12,9 | 13,2 |
| Cotton mask | Normal | 40 | 30,9 | 30,5 | 30,7 |
| Dusting cloth mask | Normal | 40 | 17,4 | 18,0 | 17,1 |
| Cotton + dusting mask | Normal | 40 | 13,0 | 12,5 | 12,9 |
| Distancing alone | Normal | 80 | 9,9 | 9,8 | 9,7 |
| Surgical mask | Normal | 80 | 3,8 | 3,1 | 4,2 |
| FFP1 | Normal | 80 | 1,0 | 1,0 | 1,0 |
| FFP2 | Normal | 80 | 1,0 | 1,0 | 1,0 |
| FFP3 | Normal | 80 | 1,0 | 1,0 | 1,0 |
| FFP2 + Surgical mask | Normal | 80 | 1,0 | 1,0 | 1,0 |
| Malpositioned FFP2 | Normal | 80 | 3,1 | 1,0 | 2,8 |
| Cotton mask | Normal | 80 | 8,0 | 7,6 | 8,2 |
| Dusting cloth mask | Normal | 80 | 4,3 | 4,0 | 4,6 |
| Cotton + dusting mask | Normal | 80 | 1,0 | 1,0 | 1,0 |
| Distancing alone | Normal | 120 | 3,9 | 3,3 | 3,6 |
| Surgical mask | Normal | 120 | 1,0 | 1,0 | 1,0 |
| FFP1 | Normal | 120 | 1,0 | 1,0 | 1,0 |
| FFP2 | Normal | 120 | 1,0 | 1,0 | 1,0 |
| FFP3 | Normal | 120 | 1,0 | 1,0 | 1,0 |
| FFP2 + Surgical mask | Normal | 120 | 1,0 | 1,0 | 1,0 |
| Malpositioned FFP2 | Normal | 120 | 1,0 | 1,0 | 1,0 |
| Cotton mask | Normal | 120 | 1,0 | 1,0 | 1,0 |
| Dusting cloth mask | Normal | 120 | 1,0 | 1,0 | 1,0 |
| Cotton + dusting mask | Normal | 120 | 1,0 | 1,0 | 1,0 |
| Distancing alone | Polypneic | 40 | 61,5 | 61,0 | 61,9 |
| Surgical mask | Polypneic | 40 | 40,0 | 40,6 | 40,4 |
| FFP1 | Polypneic | 40 | 38,9 | 39,3 | 39,2 |
| FFP2 | Polypneic | 40 | 2,8 | 3,2 | 1,0 |
| FFP3 | Polypneic | 40 | 1,0 | 1,0 | 1,0 |
| FFP2 + Surgical mask | Polypneic | 40 | 1,0 | 1,0 | 1,0 |
| Malpositioned FFP2 | Polypneic | 40 | 36,6 | 37,2 | 37,0 |
| Cotton mask | Polypneic | 40 | 41,1 | 40,8 | 41,4 |
| Dusting cloth mask | Polypneic | 40 | 29,3 | 29,8 | 29,2 |
| Cotton + dusting mask | Polypneic | 40 | 26,2 | 27,5 | 26,6 |
| Distancing alone | Polypneic | 80 | 18,6 | 19,2 | 19,1 |
| Surgical mask | Polypneic | 80 | 18,3 | 17,6 | 17,2 |
| FFP1 | Polypneic | 80 | 5,8 | 6,1 | 5,7 |
| FFP2 | Polypneic | 80 | 1,0 | 1,0 | 1,0 |
| FFP3 | Polypneic | 80 | 1,0 | 1,0 | 1,0 |
| FFP2 + Surgical mask | Polypneic | 80 | 1,0 | 1,0 | 1,0 |
| Malpositioned FFP2 | Polypneic | 80 | 7,4 | 6,8 | 6,4 |
| Cotton mask | Polypneic | 80 | 17,5 | 16,9 | 17,2 |
| Dusting cloth mask | Polypneic | 80 | 12,1 | 12,6 | 11,8 |
| Cotton + dusting mask | Polypneic | 80 | 3,4 | 6,8 | 1,0 |
| Distancing alone | Polypneic | 120 | 4,2 | 4,0 | 4,5 |
| Surgical mask | Polypneic | 120 | 3,2 | 3,1 | 2,9 |
| FFP1 | Polypneic | 120 | 1,0 | 1,0 | 1,0 |
| FFP2 | Polypneic | 120 | 1,0 | 1,0 | 1,0 |
| FFP3 | Polypneic | 120 | 1,0 | 1,0 | 1,0 |
| FFP2 + Surgical mask | Polypneic | 120 | 1,0 | 1,0 | 1,0 |
| Malpositioned FFP2 | Polypneic | 120 | 1,0 | 1,0 | 1,0 |
| Cotton mask | Polypneic | 120 | 2,8 | 3,2 | 1,0 |
| Dusting cloth mask | Polypneic | 120 | 1,0 | 1,0 | 1,0 |
| Cotton + dusting mask | Polypneic | 120 | 1,0 | 1,0 | 1,0 |
