# Supplementary material for: Effects of distancing and pattern of breathing on the filtering capability of commercial and custom-made facial masks: An in-vitro study
Source: PLoS One. 2021 Apr 22;16(4):e0250432. doi: 10.1371/journal.pone.0250432 (PMC8062003; doi:10.1371/journal.pone.0250432)

PLOS ONE

Effects of distancing and pattern of breathing on the filtering capability of commercial and custom-made facial masks: an “in-vitro” study.

--Manuscript Draft--

**Manuscript Number:**	PONE-D-20-40104
**Article Type:**	Research Article
**Full Title:**	Effects of distancing and pattern of breathing on the filtering capability of commercial and custom-made facial masks: an “in-vitro” study.
**Short Title:**	Filtering capability of facial masks
**Corresponding Author:**	Stefano Alberti<br>Universita degli Studi di Genova<br>Genova, ITALY
**Keywords:**	Facial Mask; Droplet; Viral Transmission
**Abstract:**	BACKGROUND: Since the beginning of the coronavirus disease 2019 (COVID-19) pandemics masking policies have been advocated for the general population. While the role of this measure in preventing transmission to other individuals is well known, it is unclear to which extent simpler facial masks can protect the user from inhalation. The present study aims to compare in-vitro different commercial and custom-made facial masks at different distances between the emitting source and the mask with two breathing patterns.<br>METHODS: Masks were placed on a head mannequin connected to a lung simulator, using a collecting filter placed after the mannequin airway. Certified, commercial and custom-made masks were tested at three different distances between the emitter and the mannequin: 40 cm, 80 cm and 120 cm. Two patterns of breathing were used, simulating normal and polypneic respiration, as they were found to affect even certified masks' efficacy. A solution of methylene blue was nebulized with a jet nebulizer and the percent inhaled fraction at different mask-distance-breathing pattern combinations was assessed using ultraviolet-visible spectrophotometry.<br>FINDINGS: We observed a significant effect of distance ($p < 0.001$), pattern of breathing ($p < 0.001$) and type of mask ($p < 0.001$) on inhaled fraction. All masks resulted in a lower inhaled fraction compared to simulated breathing without mask ($p < 0.001$ in all comparisons), ranging from 41.1% ± 0.3% obtained with a cotton mask at 40 cm distance with polypneic pattern to <1% for certified FFP3 and the combination of FFP2 + surgical mask at all distances and both breathing pattern conditions.<br>INTERPRETATION: Distance, type of device and breathing pattern affected the protective efficacy of masks. While the use of all types of masks resulted relevantly less inhalation compared to distancing alone (unprotected and for both breathing modes), only high-grade certified devices (FFP3 and the combination of FFP2 + surgical mask) ensured negligible inhaled fraction in all tested conditions.
**Order of Authors:**	Lorenzo Ball
	Stefano Alberti
	Claudio Belfortini
	Chiara Almondo
	Chiara Robba
	Denise Battaglini
	Carlo Cravero
	Paolo Pelosi
	Valentina Caratto
	Maurizio Ferretti
**Additional Information:**	

Powered by Editorial Manager® and ProduXion Manager® from Aries Systems Corporation

Question	Response
**Financial Disclosure**<br><br>Enter a financial disclosure statement that describes the sources of funding for the work included in this submission. Review the submission guidelines for detailed requirements. View published research articles from *PLOS ONE* for specific examples.<br><br>This statement is required for submission and **will appear in the published article** if the submission is accepted. Please make sure it is accurate.<br><br>**Unfunded studies**<br>Enter: *The author(s) received no specific funding for this work.*<br><br>**Funded studies**<br>Enter a statement with the following details:<br>• Initials of the authors who received each award<br>• Grant numbers awarded to each author<br>• The full name of each funder<br>• URL of each funder website<br>• Did the sponsors or funders play any role in the study design, data collection and analysis, decision to publish, or preparation of the manuscript?<br>• **NO** - Include this sentence at the end of your statement: *The funders had no role in study design, data collection and analysis, decision to publish, or preparation of the manuscript.*<br>• **YES** - Specify the role(s) played.<br><br>* typeset	The authors received no specific funding for this work
**Competing Interests**<br><br>Use the instructions below to enter a competing interest statement for this submission. On behalf of all authors, disclose any competing interests that could be perceived to bias this work—acknowledging all financial support and any other relevant financial or non-financial competing interests.<br><br>This statement **will appear in the published article** if the submission is	The authors have declared that no competing interests exist.

accepted. Please make sure it is accurate. View published research articles from *PLOS ONE* for specific examples.<br><br>**NO authors have competing interests**<br><br>Enter: *The authors have declared that no competing interests exist.*<br><br>**Authors with competing interests**<br><br>Enter competing interest details beginning with this statement:<br><br>*I have read the journal's policy and the authors of this manuscript have the following competing interests: [insert competing interests here]*<br><br>* typeset	
**Ethics Statement**<br><br>Enter an ethics statement for this submission. This statement is required if the study involved:<br><br>• Human participants<br>• Human specimens or tissue<br>• Vertebrate animals or cephalopods<br>• Vertebrate embryos or tissues<br>• Field research<br><br>Write "N/A" if the submission does not require an ethics statement.<br><br>General guidance is provided below. Consult the submission guidelines for detailed instructions. **Make sure that all information entered here is included in the Methods section of the manuscript.**	"N/A"

Powered by Editorial Manager® and ProduXion Manager® from Aries Systems Corporation

**Format for specific study types**<br><br>**Human Subject Research (involving human participants and/or tissue)**<br>• Give the name of the institutional review board or ethics committee that approved the study<br>• Include the approval number and/or a statement indicating approval of this research<br>• Indicate the form of consent obtained (written/oral) or the reason that consent was not obtained (e.g. the data were analyzed anonymously)<br><br>**Animal Research (involving vertebrate animals, embryos or tissues)**<br>• Provide the name of the Institutional Animal Care and Use Committee (IACUC) or other relevant ethics board that reviewed the study protocol, and indicate whether they approved this research or granted a formal waiver of ethical approval<br>• Include an approval number if one was obtained<br>• If the study involved *non-human primates*, add *additional details* about animal welfare and steps taken to ameliorate suffering<br>• If anesthesia, euthanasia, or any kind of animal sacrifice is part of the study, include briefly which substances and/or methods were applied<br><br>**Field Research**<br><br>Include the following details if this study involves the collection of plant, animal, or other materials from a natural setting:<br>• Field permit number<br>• Name of the institution or relevant body that granted permission	
**Data Availability**<br><br>Authors are required to make all data underlying the findings described fully available, without restriction, and from the time of publication. PLOS allows rare exceptions to address legal and ethical concerns. See the PLOS Data Policy and FAQ for detailed information.	Yes - all data are fully available without restriction

Powered by Editorial Manager® and ProduXion Manager® from Aries Systems Corporation

A Data Availability Statement describing where the data can be found is required at submission. Your answers to this question constitute the Data Availability Statement and **will be published in the article**, if accepted.<br><br>**Important:** Stating 'data available on request from the author' is not sufficient. If your data are only available upon request, select 'No' for the first question and explain your exceptional situation in the text box.<br><br>Do the authors confirm that all data underlying the findings described in their manuscript are fully available without restriction?	
**Describe where the data may be found in full sentences. If you are copying our sample text, replace any instances of XXX with the appropriate details.**<br><br>• If the data are **held or will be held in a public repository**, include URLs, accession numbers or DOIs. If this information will only be available after acceptance, indicate this by ticking the box below. For example: *All XXX files are available from the XXX database (accession number(s) XXX, XXX.*).<br>• If the data are all contained **within the manuscript and/or Supporting Information files**, enter the following: *All relevant data are within the manuscript and its Supporting Information files.*<br>• If neither of these applies but you are able to provide **details of access elsewhere**, with or without limitations, please do so. For example:<br><br>*Data cannot be shared publicly because of [XXX]. Data are available from the XXX Institutional Data Access / Ethics Committee (contact via XXX) for researchers who meet the criteria for access to confidential data.*<br><br>*The data underlying the results presented in the study are available from (include the name of the third party*	All relevant data are within the manuscript and its Supporting Information files.

and contact information or URL).<br>• This text is appropriate if the data are owned by a third party and authors do not have permission to share the data.<br><br>* typeset	
Additional data availability information:	

Powered by Editorial Manager® and ProduXion Manager® from Aries Systems Corporation

Manuscript Click here to access/download;Manuscript;Manuscript.docx

Full Title:

Effects of distancing and pattern of breathing on the filtering capability of commercial and custom-made facial masks: an “in-vitro” study.

Lorenzo Ball[1], Stefano Alberti[2], Claudio Belfortini[2], Chiara Almondo[1], Chiara Robba[1], Denise Battaglini[1], Carlo Cravero[3], Paolo Pelosi[1], Valentina Caratto[2] and Maurizio Ferretti[2]

University of Genoa, Genova, Italy

[1]Department of Surgical Sciences and Integrated Diagnostics

[2]Department of Chemistry and Industrial Chemistry

[3]Department of Mechanical, Energetic, Management and Transports Engineering

Short title: Filtering capability of facial masks

Corresponding author: Dr. Stefano Alberti, Department of Chemistry and Industrial Chemistry, University of Genoa, Genoa, Italy

Email: stefano.alberti@edu.unige.it

ABSTRACT

BACKGROUND: Since the beginning of the coronavirus disease 2019 (COVID-19) pandemics masking policies have been advocated for the general population. While the role of this measure in preventing transmission to other individuals is well known, it is unclear to which extent simpler facial masks can protect the user from inhalation. The present study aims to compare in-vitro different commercial and custom-made facial masks at different distances between the emitting source and the mask with two breathing patterns.

METHODS: Masks were placed on a head mannequin connected to a lung simulator, using a collecting filter placed after the mannequin airway. Certified, commercial and custom-made masks were tested at three different distances between the emitter and the mannequin: 40 cm, 80 cm and 120 cm. Two patterns of breathing were used, simulating normal and polypneic respiration, as they were found to affect even certified masks' efficacy. A solution of methylene blue was nebulized with a jet nebulizer and the percent inhaled fraction at different mask-distance-breathing pattern combinations was assessed using ultraviolet-visible spectrophotometry.

FINDINGS: We observed a significant effect of distance ($p < 0.001$), pattern of breathing ($p < 0.001$) and type of mask ($p < 0.001$) on inhaled fraction. All masks resulted in a lower inhaled fraction compared to simulated breathing without mask ($p < 0.001$ in all comparisons), ranging from 41.1% ± 0.3% obtained with a cotton mask at 40 cm distance with polypneic pattern to <1% for certified FFP3 and the combination of FFP2 + surgical mask at all distances and both breathing pattern conditions.

INTERPRETATION: Distance, type of device and breathing pattern affected the protective efficacy of masks. While the use of all types of masks resulted relevantly less inhalation compared to distancing alone (unprotected and for both breathing

modes), only high-grade certified devices (FFP3 and the combination of FFP2 + surgical mask) ensured negligible inhaled fraction in all tested conditions.

Keywords: Facial Mask, Droplet, Viral Transmission

INTRODUCTION

Since December 2019, the pandemic of coronavirus disease (COVID-19), sustained by the severe acute respiratory syndrome coronavirus-2 (SARS-CoV-2) had a dramatic impact on healthcare systems as well as people's lifestyle and social behavior[1]. This disease is transmitted through several routes, but the respiratory route is the principal one. Half of patients 2019-nCoV RNA was detected, thus concluding that saliva droplets and water aerosol may actually bring living virus [2]. In fact, the disease is mainly transmitted through the emission of droplets during coughing and sneezing, while the airborne route is more debated [3]. The distinction between these two routes is based on the size of emitted particles, with a cut-off diameter commonly set at 5 µm: droplets which are larger than 5 µm contain a large amount of virus but can spread for a shorter distance, while aerosols of smaller size could remain in the ambient air in suspension resulting in contagious aerosols [3], [4]. Also normal speaking may cause airborne virus transmission in confined environments, where small droplets likely play a major role [5].

Shortage of personal protection equipment (PPE) has heavily affected the healthcare systems in the early phases of the pandemics and have led to the public use of a variety of solutions which are generally of unknown efficiency [6]. Masks reduce the spread and transmission of respiratory particles and droplets potentially containing viruses and their use in the general population has been adopted in most countries, even though still under debate [7], [8]. In general, while the gold standard for personal protection is represented by certified respirators [9], their high cost and low availability brought interest towards cheaper alternatives such as surgical masks as well as custom-made protection devices, especially for non-healthcare workers and general population; despite the characterization of their efficacy could represent a

significant knowledge for medical professionals working in areas with a low but still real risk of contamination. The National Institute for Occupational Safety and Health defined N95 and N99 standards in the United States and the European Committee for Standardization specified with the EN 143 regulation standards for type 1, 2 and 3 filtering face pieces (FFP1, FFP2 and FFP3). While there is an undoubted role of facial masks as mean to reduce the spread of aerosols and droplets from unaware infected patients, it is unclear whether non-PPE devices can offer some degree of protection to healthy individuals [10], [11].

We aimed to compare the filtering capabilities of commercial and custom-made facial masks in an in-vitro model of mixed airborne-droplet aerial transmission. We hypothesized that the distance from the emitter and the pattern of breathing could affect the protection offered to a model of spontaneously breathing adult by the different types of investigated devices, as it was already known that a difference between certified and non-certified devices subsisted although not measured.

METHODS

Experimental model

As illustrated in **Figure 1**, the emitter was simulated using a Hudson Micromist (Teleflex, US) small volume jet nebulizer operated at 7 L/m driving flow. Under these conditions, the nebulizer generates a distribution of drops with a median size of 5 µm and an asymmetric distribution [12] thus covering the range of both airborne and droplet transmission. As marker, we nebulized a solution of 3.44 g/L of methylene blue. The receiver was simulated with a pneumatic lung simulator (Dimar, Mirandola, Italy) connected to a head mannequin to simulate an exposed individual, protected with different masks. We simulated two respiratory patterns: normal breathing with 550 mL

tidal volume and respiratory rate of 14 min^{-1}, and mildly polypneic pattern with 550 mL tidal volume and respiratory rate of 20 min^{-1}.

The emitter was placed in front of the receiver at three distances: 40, 80 and 120 cm. Between the receiver and the lung simulator, a custom-made filter with disposable cotton pads, as described in a previous study [13], was placed to intercept the nebulized methylene blue. The nebulizer was operated for 40 min loaded with 16 ml of methylene blue solution refilling the nebulizer to its maximum capacity during brief interruptions of nebulization, and each mask-distance-breathing pattern combination was replicated three times. As detailed in **Table 1**, we tested certified PPEs, commercially available non-PPE masks and custom-made devices and compared them to the effect of distancing alone, without mask. **Figure 2** shows the tested masks, non-PPEs and custom-made devices in all examined configurations (i.e., alone and their combinations): Surgical mask, FFP1, FFP2, FFP3, FFP2 + Surgical mask, Malpositioned FFP2 (uncovered nose), Cotton mask, Dusting Cloth mask and Cotton + Dusting mask.

Figure 1: Experimental setting.

Figure 2: Tested devices.

Table 1: Detailed description of the different types of tested devices, according to their certification, commercial name and manufacturer

Short name	Type of device	Description	Commercial name	Manufacturer
Surgical mask	Commercial	Multilayer surgical mask	Non-woven face mask	Sinomedic, Serbia
FFP1*	Commercial, certified	PPE certified as FFP1	Shell Mask XMASC	Icoguanti S.p.A., Italy
FFP2*	Commercial, certified	PPE certified as FFP2	Aura 9320+	3M, Minnesota, US

FFP3*	Commercial, certified	PPE certified as FFP3	2737 FFP3 RD	GVS Filter Technology, UK
FFP2* + Surgical mask	Combination of two devices	FFP2 mask plus a covering surgical mask		
Malpositioned (uncovered nose and covered mouth) FFP2*	Commercial, certified, malpositioned	FFP2 placed not optimizing fitting and leaving a small space between nose and mask		
Cotton mask	Commercial, not certified	Two-layers cotton plus non-woven tissue mask		
Dusting cloth mask	Custom-made	Two layers of dusting cloth shaped as face mask	Swiffer	Procter & Gamble, US
Cotton + dusting mask	Combination of two devices	Cotton mask plus a covering dusting mask		

*FFP stands for Filtering Factor Protection of type 1,2 and 3, according to EN 143 regulations standards, as depicted in Introduction paragraph.

Inhaled fraction measurement

The amount of methylene blue deposed on the cotton pads was analyzed with ultraviolet-visible spectrophotometry (Lambda 35, Perkin Elmer, US), operated at a wavelength of 664 nm, corresponding to the minimum transmittance of methylene blue [14]. We compared the amount detected at each mask-distance-breathing pattern combination with the amount detected with the emitter placed directly in contact with the mannequin airway opening, considered as reference (100% inhaled fraction). A blank cotton pad was used as reference for 0% inhaled fraction. The filtering capability was expressed as the percent inhaled fraction of methylene blue compared to zero-distance and no mask with the two patterns of breathing. We assessed the minimum amount of detectable inhaled fraction (*i.e.*, sensitivity) in a calibration experiment in which nebulization time was decreased in 1 s steps. The minimum nebulization time required to detect a non-zero transmittance was 22 s, corresponding to 0.92% of the nebulization time used in our experimental runs. We therefore assumed a sensitivity

of 1%; when the measured inhaled fraction was below this threshold, we assumed an inhaled fraction of 1%.

Statistical analysis

The filtering capability was reported as percent inhaled fraction compared to distancing alone and aggregated as average ± standard deviation. We used a linear model to investigate the effects of distance, device and pattern of breathing on inhaled fraction. We used the Dunnett test for post-hoc multiple comparisons. All analyses were performed with SPSS 25.0 (IBM, Chicago, Illinois). Statistical significance was considered for two–tailed $p < 0.05$.

RESULTS

Figure 3 reports the percent filtering capability at 40 cm, 80 cm and 120 cm with all the tested devices and the two patterns of breathing, compared to unprotected distancing alone. We observed a significant effect of distance ($p < 0.001$), pattern of breathing ($p < 0.001$) and type of mask ($p < 0.001$) on the inhaled fraction. Indeed, all masks resulted in an inhaled fraction lower than that without mask ($p < 0.001$ in all comparisons), ranging from 41.1% ± 0.3% obtained with a cotton mask at 40 cm distance with polypneic pattern to <1% for certified FFP3 and the combination of FFP2 + surgical mask at all distance and breathing pattern conditions. At any distance, all devices had higher filtering capability than distancing alone and the magnitude of differences between devices was higher at closer distances. The pattern of breathing had a negative impact on the filtering capacity: compared to normal breathing, the polypneic pattern of breathing decreased the filtering capability of all devices except FFP1, FFP2 and FFP3 masks at 40 cm and 80 cm, while at 120 cm we did not observe differences between patterns except for the surgical mask. Also, incorrect positioning

of the mask decreased the mask's performances. The dependence of the percent inhaled fraction as a function of the distance and breathing pattern is shown in **Figure 4**. The polypneic breathing configuration negatively affected all masks' performances except FFP2+Surgical and FFP3.

Figure 3: Effects of different distances and pattern of breathing on the inhaled fraction with the tested masks during normal (white bars) and polypneic (gray bars) pattern of breathing. The inhaled fraction is expressed as percent of the amount inhaled without mask and at zero distance from the emitter. Bars represent means, error bars the standard deviation. The horizontal gray bar represents the sensitivity limit of our technique. *Significant difference compared to distancing alone ($p<0.001$), °Significant difference compared to distance of 40 cm, § significant difference compared to normal pattern of breathing at same distance ($p<0.01$).

Figure 4: Effect of distance from the emitter on inhaled fraction with different masks during normal (A) and polypneic (B) pattern of breathing. *Significant difference compared to distancing alone ($p<0.001$).

DISCUSSION

The main findings of this study are that: 1) all investigated variables resulted significant ($p < 0.001$); 2) all tested masks reduced the inhaled fraction compared to distancing alone; 3) distance decreased the inhaled fraction; 4) the differences between devices were more pronounced at shorter distances; 5) polypneic breathing pattern led to a decrease in most masks' efficacy; 6) findings clarified the limitations/advantages of using masks both for individual and other protection.

The determination of masks' efficacy is a complex topic that still represents an active field of research but in the present work, we developed an inexpensive method to test the filtering capabilities of several face masks, simulating different significant scenarios affecting their efficiency. In a recent in-vitro study, authors demonstrated that several custom-made masks approached the performances of surgical masks [6]. However, in that study authors focused on the masks' capability of filtering expelled droplets, thus the efficacy of masks in protecting other individuals, while in this study we focused on the inhaled fraction, i.e. on the ability of masks to protect the individual from an unknown, unmasked subject.

We investigated the effect of different type of devices considering distance and type of pattern of breathing as factors modifying mask efficacy. The jet nebulizer guaranteed a distribution of nebulized particles covering the size range of both droplet and airborne transmission models. While the main route of transmission of COVID-19 seems to be related to larger droplets, smaller drops emitted during normal speaking can survive in suspension in the air, resulting in airborne transmission[15]. Direct airborne transmission due to coughing or sneezing is a complex phenomenon: bigger particles may remain in the surrounding air for several minutes prior to dropping to the ground; while smaller particles tend to remain into the air, thus favoring long-distance diffusion. In case of absent ambient ventilation, these particles will slowly and steadily diffuse throughout the space, remaining in the air for many hours [16]. Particularly, a single sneeze can generate about 40,000 large droplets; and most of them will undergo immediately division into smaller droplets, accounting an 80% of the particles being smaller than 100 micrometers. Since coughing is more frequent than sneezing, this represents the most common route for airborne transmission. These mechanisms

should be carefully accounted for in this pandemic frame, as they play a crucial role for understanding the airborne transmission of infectious diseases [17], [18].

Considering a short distance as in the 40 cm experimental setting, a typical distance between an infected patient and a healthcare professional or occurring during close social interaction, the only PPE that offered high degree of protection according to our findings were FFP2, FFP3 and FFP2+surgical mask. Moreover, incorrect positioning of the FFP2 mask resulted in a relevant decrease of its filtering capability. Custom made devices based on cotton, dusting cloth and their combinations offered low protection but comparable to that obtained with a surgical or FFP1 mask. Distance alone markedly reduced the inhaled fraction also without masks, thus underlining how physical distancing policies intrinsically increase the protective effect of all other measures. In fact, at 120 cm from the emitting source, both breathing patterns resulted in an inhaled fraction below 5% also without mask and any type of mask lowered this value below the sensitivity threshold of our technique with a quiet pattern of breathing. On the other hand, at the same distance but with a polypneic pattern, only FFP2, FFP3 and dusting cloth custom-made devices ensured inhaled fraction below 1%. However, also in the worst experimental condition at close distance and with polypneic breathing, also cheap non-certified and custom-made devices reduced the inhaled fraction to a range from 41% to less than 1%, compared to more than 60% obtained without mask.

These findings are relevant for the multi-use nature of custom-made and low-cost devices, which could guarantee a higher supply as well as a lower disposal and production of waste. Within this pandemic context, universal face masking policies might help in reducing the severity of disease. In fact, sub-optimal protection could result in lower inhaled viral load, possibly resulting in a larger percentage of mild or asymptomatic new infections[19]. This in-vitro study adds to the current knowledge as

it explores the hypothesis that, in addition to preventing dispersion of droplets, non-certified devices might anyway offer a low but significant reduction of the inhaled fraction. Previous epidemiologic investigations have suggested a strong relationship between public masking and pandemic control, reducing the growth of the epidemic curve [19]. Our findings suggest that this might also be related to some degree of individual protection, in addition to the known ability of protecting others through reduction of emitted droplets, therefore they may even be used by healthcare workers operating in areas with a small but still existing hazard of contamination. This study has limitations that should be addressed. First, it is an in-vitro study, thus we were not able to conclude on actual potential of preventing infections. However, we simulated a distribution of droplets similar to that achieved in the real world, and we tested realistic scenarios and different devices. Second, our technique had a sensitivity of around 1%, insufficient to analyze the differences between highly efficient devices. However, the method was inexpensive and highly reproducible, and sufficiently sensitive to analyze differences between lower-grade devices. Third, we did not investigate the effects of additional factors and devices, such as face shields. However, while of proven efficacy in the healthcare setting [20], their use in the general population is very limited.

Conclusions

Distance, type of device and breathing pattern affected the protective efficacy of masks. While only high-grade certified devices ensured negligible inhaled fraction in all conditions, the use of all types of masks resulted relevantly less inhalation compared to distancing alone. These finding support the concept that, in addition to the protective effect on emission toward other individuals, universal masking policies

could provide protection also to the subject wearing the mask, even in the case of low-cost devices.

DECLARATIONS:

Ethical approval and consent to participate

Not applicable.

Consent for publication

Not applicable.

Availability of data and materials

The authors will share the dataset on reasonable request

Competing interests

None

Funding

Institutional funding only

Authors' contributions

VC, LB, MF, PP, CC designed the study. SA, CA, VC, CB performed the experiments. SA, LB, CR, DB analyzed the data. PP, LB, MF interpreted the results. SA, VC, LB, DB drafted the manuscript. All authors approved the final version of the manuscript.

Acknowledgements

None

REFERENCES

[1] L.-L. Ren *et al.*, “Identification of a novel coronavirus causing severe pneumonia in human: a descriptive study,” *Chin. Med. J. (Engl.)*, vol. 133, no. 9, pp. 1015–1024, May 2020, doi: 10.1097/CM9.0000000000000722.

[2] W. Zhang *et al.*, “Molecular and serological investigation of 2019-nCoV infected patients: implication of multiple shedding routes,” *Emerg. Microbes Infect.*, vol. 9, no. 1, pp. 386–389, Jan. 2020, doi: 10.1080/22221751.2020.1729071.

[3] P. Anfinrud, V. Stadnytskyi, C. E. Bax, and A. Bax, “Visualizing Speech-Generated Oral Fluid Droplets with Laser Light Scattering,” *N. Engl. J. Med.*, vol. 382, no. 21, pp. 2061–2063, May 2020, doi: 10.1056/NEJMc2007800.

[4] N. van Doremalen *et al.*, “Aerosol and Surface Stability of SARS-CoV-2 as Compared with SARS-CoV-1,” *N. Engl. J. Med.*, vol. 382, no. 16, pp. 1564–1567, Apr. 2020, doi: 10.1056/NEJMc2004973.

[5] V. Stadnytskyi, C. E. Bax, A. Bax, and P. Anfinrud, “The airborne lifetime of small speech droplets and their potential importance in SARS-CoV-2 transmission,” *Proc. Natl. Acad. Sci.*, vol. 117, no. 22, pp. 11875–11877, Jun. 2020, doi: 10.1073/pnas.2006874117.

[6] E. P. Fischer, M. C. Fischer, D. Grass, I. Henrion, W. S. Warren, and E. Westman, “Low-cost measurement of face mask efficacy for filtering expelled droplets during speech,” *Sci. Adv.*, vol. 6, no. 36, p. eabd3083, Sep. 2020, doi: 10.1126/sciadv.abd3083.

[7] Z. Zhai, “Facial mask: A necessity to beat COVID-19,” *Build. Environ.*, vol. 175, p. 106827, May 2020, doi: 10.1016/j.buildenv.2020.106827.

[8] S. Feng, C. Shen, N. Xia, W. Song, M. Fan, and B. J. Cowling, “Rational use of face masks in the COVID-19 pandemic,” *Lancet Respir. Med.*, vol. 8, no. 5, pp. 434–436, May 2020, doi: 10.1016/S2213-2600(20)30134-X.

[9] World Health Organization., “Rational use of personal protective equipment for coronavirus disease (COVID-19) and considerations during severe shortages: interim guidance.” World Health Organization, Apr. 06, 2020, [Online]. Available: https://www.who.int/publications/i/item/rational-use-of-personal-protective-equipment-for-coronavirus-disease-(covid-19)-and-considerations-during-severe-shortages.

[10] M. Liang *et al.*, “Efficacy of face mask in preventing respiratory virus transmission: A systematic review and meta-analysis,” *Travel Med. Infect. Dis.*, vol. 36, p. 101751, Jul. 2020, doi: 10.1016/j.tmaid.2020.101751.

[11] C. P. K. Wadhwani, P. S. Rosen, A. S. Rosen, Y. H. Wadhwani, and K. H. Chung, “A technique to improve the viral protection of a procedure mask in absence of an N95 shield respirator.,” *Perio-Implant Advis.*, Mar. 2020.

[12] C. J. Hallberg, M. Lysaught, C. E. Zmudka, W. K. Kopesky, and L. E. Olson, “Characterization of a human powered nebulizer compressor for resource poor settings,” *Biomed. Eng. OnLine*, vol. 13, no. 1, p. 77, 2014, doi: 10.1186/1475-925X-13-77.

[13] L. Ball *et al.*, “Effects of Nebulizer Position, Gas Flow, and CPAP on Aerosol Bronchodilator Delivery: An In Vitro Study,” *Respir. Care*, vol. 61, no. 3, pp. 263–268, Mar. 2016, doi: 10.4187/respcare.04275.

[14] S. Alberti, V. Caratto, D. Peddis, C. Belviso, and M. Ferretti, “Synthesis and characterization of a new photocatalyst based on TiO_2 nanoparticles supported on a magnetic zeolite obtained from iron and steel industrial waste,” *J. Alloys Compd.*, vol. 797, pp. 820–825, Aug. 2019, doi: 10.1016/j.jallcom.2019.05.098.

[15] L. Morawska *et al.*, “Size distribution and sites of origin of droplets expelled from the human respiratory tract during expiratory activities,” *J. Aerosol Sci.*, vol. 40, no. 3, pp. 256–269, Mar. 2009, doi: 10.1016/j.jaerosci.2008.11.002.

[16] L. Brosseau, “COVID-19 transmission messages should hinge on science.,” *Center for Infectious Disease Research and Policy*, Mar. 16, 2020. http://www.cidrap.umn.edu/news-perspective/2020/03/commentary-covid-19-transmission-messages-should-hinge-science.

[17] A. Fernstrom and M. Goldblatt, “Aerobiology and Its Role in the Transmission of Infectious Diseases,” *J. Pathog.*, vol. 2013, pp. 1–13, 2013, doi: 10.1155/2013/493960.

[18] L. Bourouiba, “Turbulent Gas Clouds and Respiratory Pathogen Emissions: Potential Implications for Reducing Transmission of COVID-19,” *JAMA*, Mar. 2020, doi: 10.1001/jama.2020.4756.

[19] M. Gandhi and G. W. Rutherford, “Facial Masking for Covid-19 — Potential for ‘Variolation’ as We Await a Vaccine,” *N. Engl. J. Med.*, vol. 383, no. 18, p. e101, Oct. 2020, doi: 10.1056/NEJMp2026913.

[20] M. E. Bhaskar and S. Arun, “SARS-CoV-2 Infection Among Community Health Workers in India Before and After Use of Face Shields,” *JAMA*, vol. 324, no. 13, p. 1348, Oct. 2020, doi: 10.1001/jama.2020.15586.

Figure 1

Click here to access/download;Figure;Figure 1.png

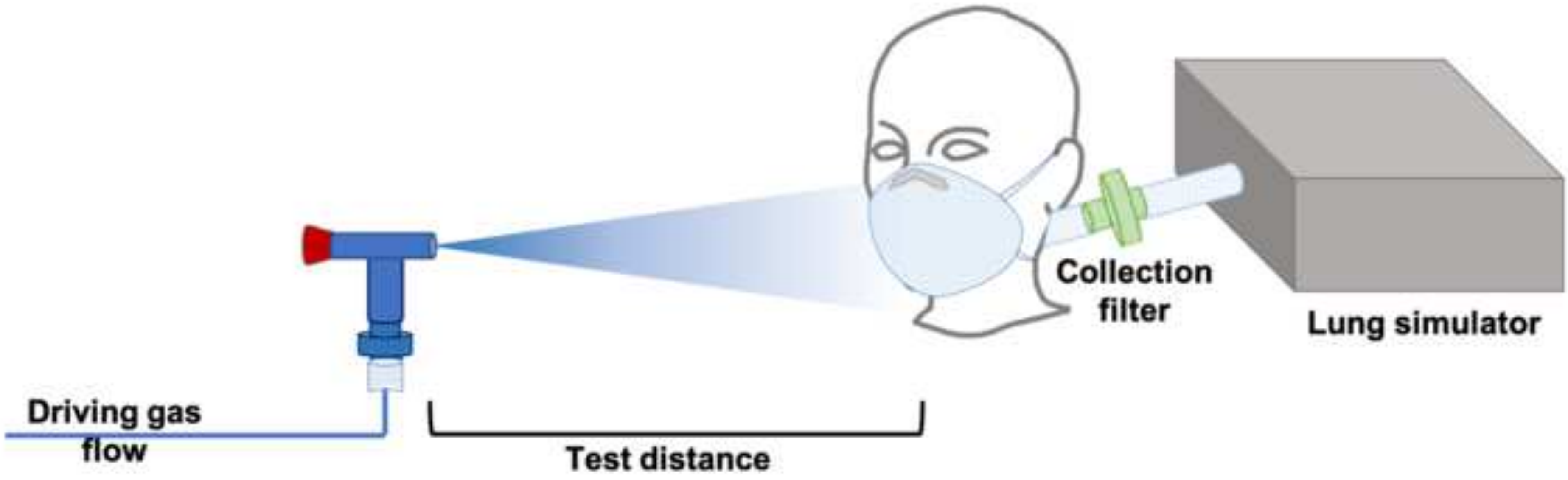

Figure 2

Click here to access/download;Figure;Figure 2.png

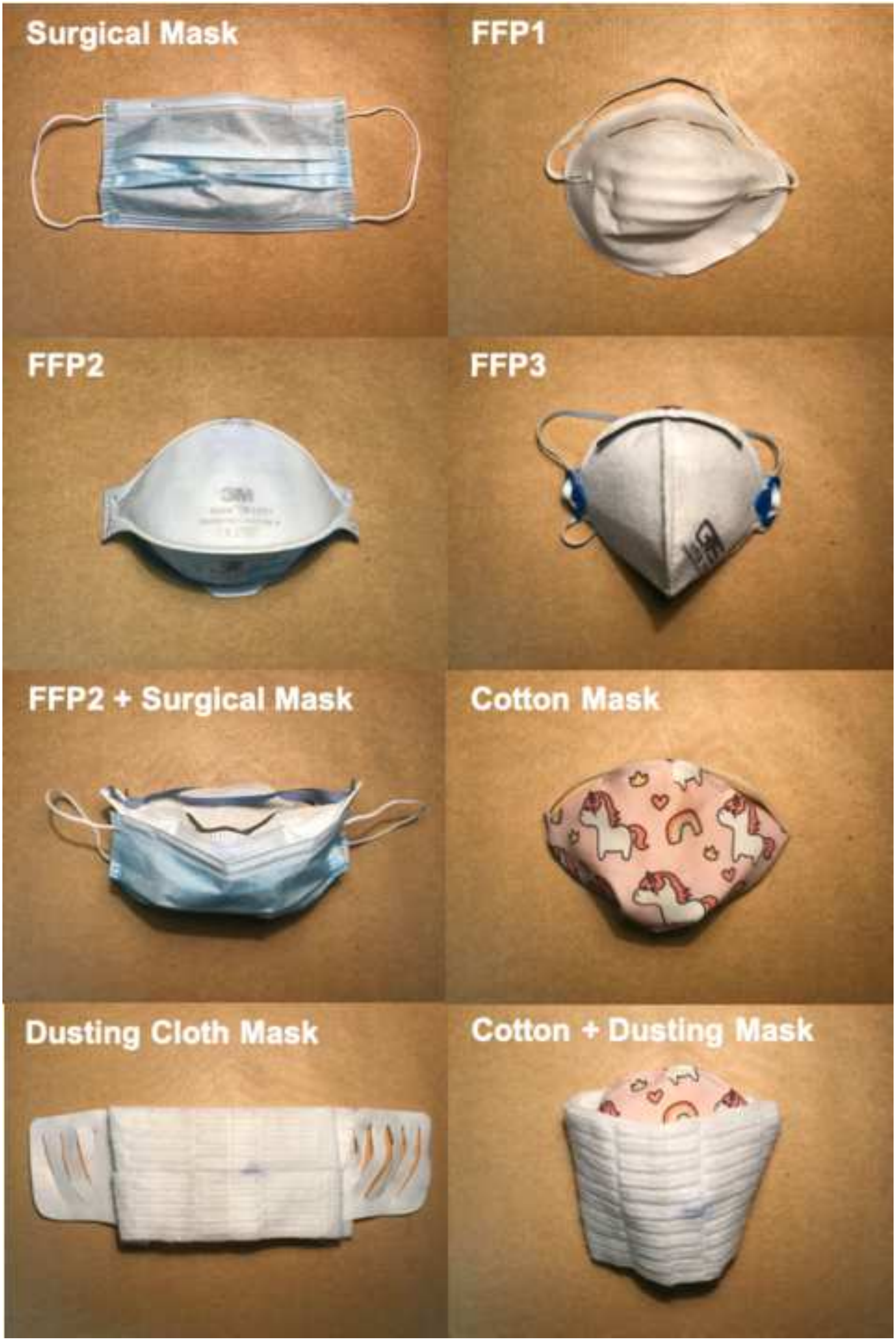

Figure 3

Click here to access/download;Figure;Figure 3.png

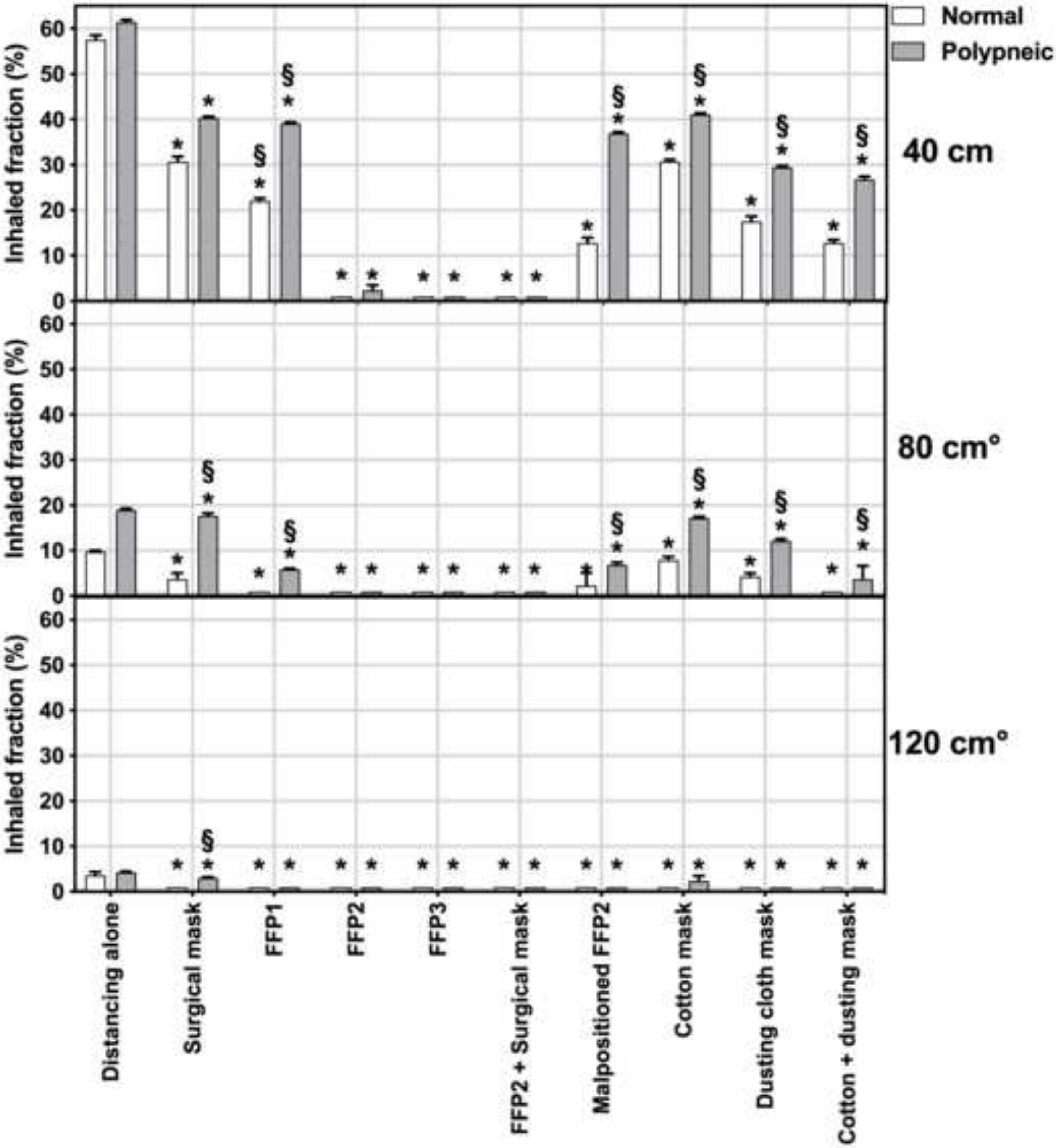

Figure 4

Click here to access/download;Figure;Figure 4.png

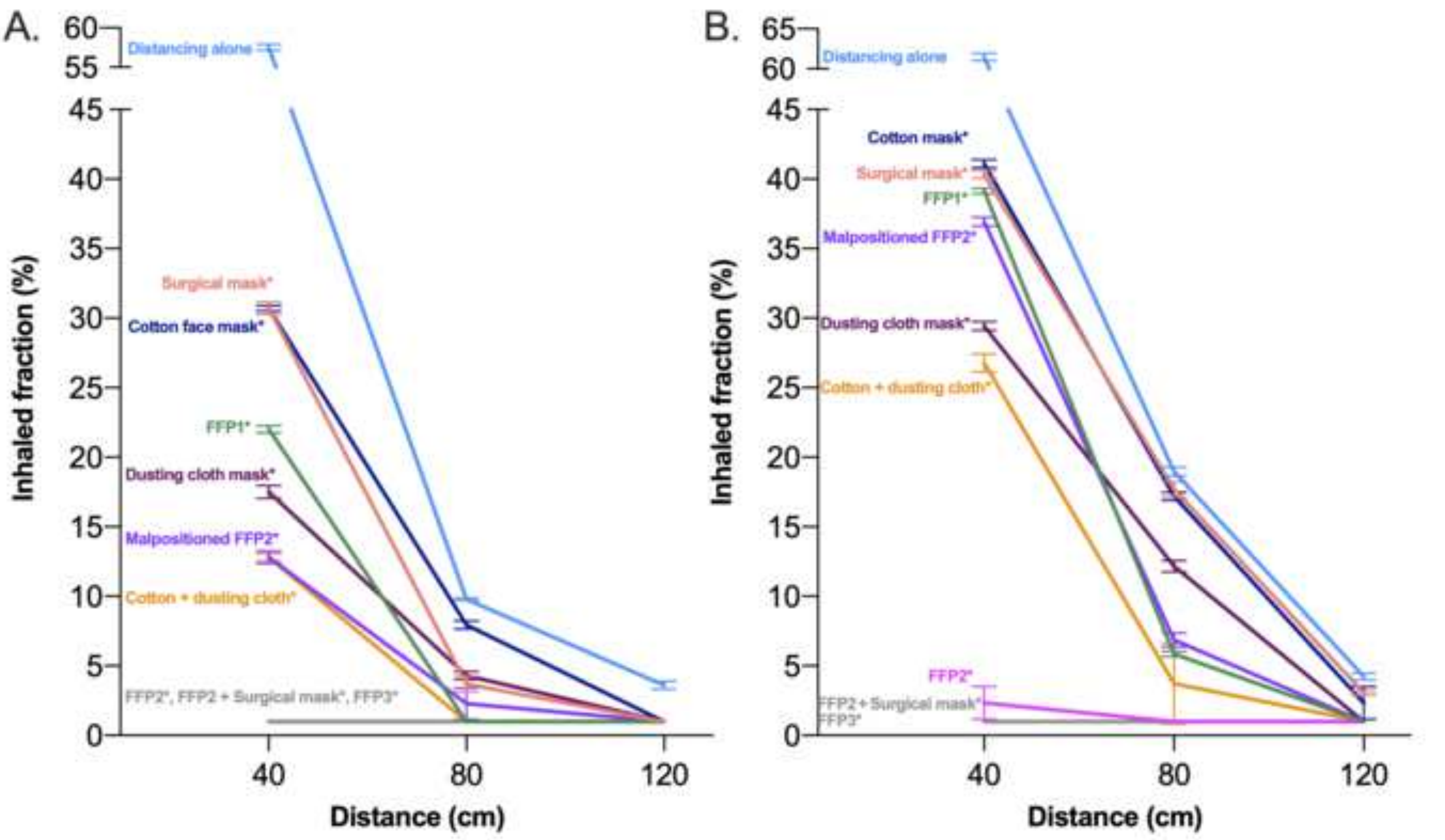

Supplement: Attachment — Submitted filename: PONE-D-20-40104_reviewerTA.pdf [file pone.0250432.s002.pdf]
